# Novel gene sets improve set-level classification of prokaryotic gene expression data

**DOI:** 10.1186/s12859-015-0786-7

**Published:** 2015-10-28

**Authors:** Matěj Holec, Ondřej Kuželka, Filip železný

**Affiliations:** Faculty of Electrical Engineering, Czech Technical University, Technická 2, Prague, 166 27 Czech Republic; School of Computer Science and Informatics, Cardiff University, Queen’s Buildings, 5 The Parade, Roath, Cardiff, CF24 3AA UK

**Keywords:** Set-level, Classification, Gene expression, Regulation, Interaction

## Abstract

**Background:**

Set-level classification of gene expression data has received significant attention recently. In this setting, high-dimensional vectors of features corresponding to genes are converted into lower-dimensional vectors of features corresponding to biologically interpretable gene sets. The dimensionality reduction brings the promise of a decreased risk of overfitting, potentially resulting in improved accuracy of the learned classifiers. However, recent empirical research has not confirmed this expectation. Here we hypothesize that the reported unfavorable classification results in the set-level framework were due to the adoption of unsuitable gene sets defined typically on the basis of the Gene ontology and the KEGG database of metabolic networks. We explore an alternative approach to defining gene sets, based on regulatory interactions, which we expect to collect genes with more correlated expression. We hypothesize that such more correlated gene sets will enable to learn more accurate classifiers.

**Methods:**

We define two families of gene sets using information on regulatory interactions, and evaluate them on phenotype-classification tasks using public prokaryotic gene expression data sets. From each of the two gene-set families, we first select the best-performing subtype. The two selected subtypes are then evaluated on independent (testing) data sets against state-of-the-art gene sets and against the conventional gene-level approach.

**Results:**

The novel gene sets are indeed more correlated than the conventional ones, and lead to significantly more accurate classifiers. The novel gene sets are indeed more correlated than the conventional ones, and lead to significantly more accurate classifiers.

**Conclusion:**

Novel gene sets defined on the basis of regulatory interactions improve set-level classification of gene expression data. The experimental scripts and other material needed to reproduce the experiments are available at http://ida.felk.cvut.cz/novelgenesets.tar.gz.

**Electronic supplementary material:**

The online version of this article (doi:10.1186/s12859-015-0786-7) contains supplementary material, which is available to authorized users.

## Background

*Set-level classification* of gene expression data has received significant attention recently [1–6]. Unlike in more conventional gene expression analysis, the set-level approach assumes that high-dimensional vectors of gene expressions are represented by lower-dimensional vectors of *aggregated* expressions. The latter are aggregated over apriori defined gene sets. The sets are specified in terms of formalized biological background knowledge; a single set may e.g. collect all genes acting in a specific metabolic pathway. In this setting, predictive classifiers are learned using the lower-dimensional set-level representation. Besides obvious benefits in the interpretability of the learned classifiers, the set-level approach is mainly motivated by the problem of high feature dimension contrasting with the low number of available samples, which has been characteristic of gene expression data analysis.

Given the entailed reduction in sample dimensionality, the set-level approach should lead to a decreased risk of overfitting potentially resulting in improved accuracy of induced predictive models. Unfortunately, this expectation was not confirmed by empirical research [1, 2, 5, 6].

In this paper we hypothesize that the lack of predictive accuracy improvements observed in the previous studies was due to the adoption of unsuitable types of gene sets. In set-level gene expression analysis (e.g., [1–3, 5]), a usual way to define prior gene sets is through the Gene Ontology [7] (GO) terms or the KEGG [8] database of metabolic pathways. In the former case, a gene set corresponds to an ontology term (representing a function, process, or a cellular component) and collects all genes annotated by that term. In the latter, a gene set contains genes whose product acts in a specific cellular pathway. This type of prior knowledge is also frequently used in tasks of gene enrichment analysis [9], gene functional clustering [4, 10], pattern mining [11, 12], as a regularization technique in machine learning [13], and also in clinical studies [14, 15].

When transforming expression data from the gene level to the lower-dimensional gene-set level, some information is obviously lost. Intuitively, this loss is minimized when the set-level representation preserves most of the variance of the original data. This happens when the defined sets aggregate genes highly correlated in terms of their expression, thus minimizing variance *inside* the sets, and maintaining variance *between* them. The GO and KEGG gene sets mentioned above do not tend to gather genes with strongly correlated expression as they are defined through properties of and functional relations among protein products of the genes rather than interactions directly pertaining to transcription regulation. This reasoning is empirically supported, for example, by an improvement of gene expression estimations using the operon structure [16] and relatively higher consistence of genes contained in the same operon as opposed to gene groups defined by a common GO term or KEGG pathway membership [17].

On the other hand, high correlation can be expected between expressions of genes which share activating regulatory proteins (transcription factors). We use two sources of available formalized knowledge to define such gene sets. One is represented by a regulation network where directed edges connect transcription factors with their gene targets. The other source, which is specific to prokaryotes, is the known operon structure of the genome. Operons are clusters of genes transcribed into an mRNA as a single unit. To harness and compare both information on operons and transcription factors, we restrict our experimental material to prokaryotic gene expression data sets.

Note that the aggregated expression of genes positively regulated by a transcription factor may be seen as a proxy for the *activity* of that transcription factor and its presence in sufficient *concentration* in the cell. Such information is obviously highly relevant to the prediction of the target phenotype, and it cannot be inferred from the microarray-measured values of expression of the transcription factor only.

The main purpose of this paper is to evaluate the performance of the newly designed gene sets in the context of predictive classification, against the gene sets used in previous work and against the conventional gene-level classification. To this end, the next section exposes the details concerning the design of the novel gene sets and other methodological ingredients of our approach. Subsequently, we address the empirical questions regarding the classification performance and the intra-set expression correlation, and then conclude the study.

## Methods

Here we first explain the proposed novel gene sets as well as the conventional gene sets used for reference in comparative experiments. Then we briefly describe the machine learning scenarios which we follow to assess the quality of the gene sets. Lastly, we describe the protocols for collecting the training data and for statistical validation.

### Gene sets

As motivated in the Background, sets of co-regulated genes should form good features for phenotype prediction.

The adjective *co-regulated* allows multiple interpretations. Here we explore various such interpretations giving rise to six different novel types of gene sets. Three of them exploit the known operon-based structure of the prokaryotic genome, for which we do not need to know the exact regulatory network. For the other three, we exploit information about the gene-gene regulatory interactions.

We will define gene sets by specifying a condition that the member genes should satisfy, e.g. genes “controlled by transcription factor T”. Without restating it explicitly, we will always consider that such sets are *maximal*, that is, *all* genes satisfying the defining condition of a set are included in the set.

#### Operon structure based gene sets

In prokaryotes, genes are organized into contiguous clusters called operons. We exploit the operon structure to define three types of gene sets.

##### Operon (OPR)

An operon is generally transcribed into messenger RNA as a single unit. Thus we can expect the expressions of genes in a single operon to be more correlated than those of randomly selected genes. Therefore the most basic type of gene sets is defined by genes located in the same operon.

Some operons may also have multiple promoters, possibly located even inside the operon, which means that sometimes only a subset of genes of the operon may be jointly transcribed. This motivates two more definitions of gene set types.

##### Transcription Unit (TU)

**Fig. 1:**
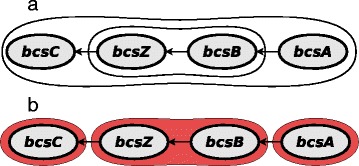
Example of operon based gene sets. **a** The operon *bcsABZC* contains genes *bcsC, bcsZ, bcsB, bcsA*, and contains transcription units *bcsABZC* and *bcsBZ*. **b** COPR sets are consecutive set of genes in operons which are always co-transcribed

A transcription unit is a group of genes transcribed from a single promoter. Unlike operons, transcription units may overlap and one transcription unit can be completely contained in another one (Fig. 1a).

TU sets include all OPR sets as well some of their subsets. Thus for some TU sets (at least for the OPR ones) again only a part of a transcription unit is transcribed into mRNA. Therefore, we also consider the following gene set type.

##### Continuous Operon Subsequence (COPR)

A COPR gene set is a maximal non-interrupted subsequence (“chunk”) of an operon, i.e. no promoter is located between any two genes contained in that subset. It follows that the expression of genes in COPR set should be highly correlated. COPR sets do not overlap.

COPR sets are maximal non-overlapping sequences of genes which divide an operon such that no TU starts or ends inside of any chunk (Fig. 1b).

#### Transcription factor based gene sets

Next we define gene sets based on gene-gene regulation interactions.

##### Transcription Factor (TF)

**Fig. 2:**
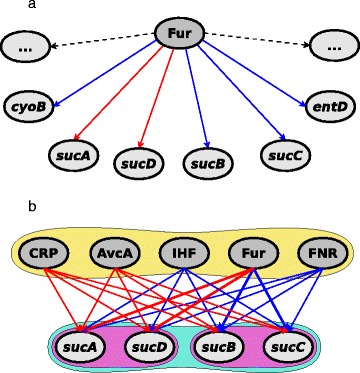
Example of transcription-factor based gene sets. **a** A transcription factor *Fur* regulates altogether 130 genes including positively regulated genes e.g., *sucA, sucD* and negatively regulated genes e.g., *cyoB, sucB, sucC, entD*. All these regulated genes constitute the gene set. **b** A complex regulon defined by genes *sucA, sucD, sucB and sucC* can be divided into two strict regulons defined by two pairs of genes *(sucA, sucD)* and *(sucB, sucC)*. All the three mentioned regulons are regulated only by a common set of transcription factors *CRP, ArcA, IHF, Fur and FNR*

The simplest of this type of gene sets are *TF gene sets* (Fig. 2a) which are composed of sets of genes having a regulating transcription factor in common. If is further assumed that the nature of the regulatory influence (activation, repression, dual or unknown) of the transcription factor is the same for all the genes in the set. Thus we can have up to four gene sets composed of genes regulated by a single transcription factor.

While the shared transcription factor in a TF set contributes to positive correlation of the expression of the member genes, this effect is naturally limited as the genes may as well be co-regulated by other transcription factors which are not shared within the set. This is a motivation for introducing two additional transcription factor based gene sets.

##### Regulon (REG)

The second of this type of gene sets are *Regulon gene sets* (Fig. 2b) which are based on the notion of *regulon* from [18]. A REG gene set consists of genes which are regulated by exactly the same set of transcription factors.

Since the type of regulatory relations is not considered in a REG set, the expressions of the member genes in it may be uncorrelated simply due to a transcription factor having a positive effect on some of the members and negative on others. The following gene set definition aims to avoid this.

##### Strict Regulon (SREG)

The third type of transcription factor based gene sets is based on *strict regulons* [19] (Fig. 2b). A strict regulon is a set of genes controlled by the same set of regulatory genes each of which must have the same role for all of the regulated genes. Thus the only difference between REG sets and SREG sets is the additional condition regarding the regulation type.

The members of all the defined gene sets were determined from the transcriptional regulation network of Escherichia coli K-12 as described in the RegulonDB (ver. 8.2) [20].

#### Baseline gene sets

##### Gene Ontology and KEGG (GO+KEGG)

In set-level gene expression analysis, a frequent way of defining prior gene sets is through the Gene Ontology [7] (GO) terms or the KEGG [8] database of metabolic pathways. In the former case, a set is defined by a GO term and collects all genes annotated by that term. In the latter case, a set pertaining to a pathway includes all genes whose product act in that pathway. The sets derived from these two conventional sources are combined into a single reference family of sets used in comparative experiments. We extracted these gene sets from the R package *Genome wide annotation for Escherichia coli strain K12* (version 2.9.0).

##### Randomized gene sets

For all the gene sets of the types defined above, we also defined their randomized counterparts. For a given gene-set type, genes in the randomized gene sets are shuffled among all gene sets of that type. Thus, the size proportions of the gene sets remain unchanged. The reason for defining such randomized controls is to isolate the effects of involving relevant background knowledge (as in the case of the genuine sets defined through biological principles) from those of plain dimensionality reduction through feature aggregation (as in the case of randomized gene sets).

Table 1 provides a summary of all the constructed gene sets and their quantitiative properties.

**Table 1:** A summary of gene-set types and their properties

Gene-set type	# sets	# genes	# of genes in set		
			Median	Mean	Max.
Operon Based					
Operon (OPR)	2649	4524	1	1.708	16
Transcriptional unit (TU)	3213	4524	1	1.685	16
Continuous subsequence (COPR)	3164	4524	1	1.430	12
Transcription Factor Based					
Transcription factor (TF)	186	1685	7	24.720	534
Regulon (REG)	459	1685	2	3.671	61
Strict regulon (SREG)	541	1685	2	3.115	51
Conventional					
GO+KEGG	260	2734	12	31.830	847

For each of the type, the smallest sets contain exactly one gene. The “# genes” column contains the number of genes included in at least one set of the given type. Since the sets are not disjoint, # genes / # sets ≠ mean. The table does not list the seven randomized gene set collections, which possess exactly the same statistics as the respective listed types except their member genes are permuted

### Machine learning

To evaluate the quality of the proposed gene sets, we performed experiments in which a machine-learning algorithm was used to learn a classifier for predicting a phenotype class from measured gene expressions. We used the *support vector machine* learning algorithm [21]. In this approach, samples are viewed as points in a vector space with coordinates given by the values of the sample’s features. A classifier is sought in the form of a hyperplane that separates training samples of distinct classes and maximizes the distance to the points nearest to the hyperplane (i.e. maximizing the margin) in that space. We used the implementation from the *R package e1071, version 1.6-1*.

In the conventional gene-level setting, features of a sample correspond to the expressions of the individual genes. In the set-level approach, however, features correspond to the pre-defined gene sets and their values for a given sample aggregate the expressions of the member genes in that sample. Thus, we need a data aggregation function to compute a single real number representing the aggregated expression of genes in a gene set. The simplest option, which we adopt here, is the arithmetic average, although other aggregation methods have been proposed in the context of set-level gene expression analysis before, such as the median value, or the set-signature (SET-SIG) method [1], which fits the aggregation function using class labels available in training data.

### Gene-expression data

Our experimental evaluation involves microarray gene expression datasets measured in the bacteria Escherichia coli. We selected this popular model organism for the following reasons. First, it is estimated that about ^2^ /_3_ of its transcriptional regulatory proteins and most of their targets are already known and described in the publicly available database RegulonDB [20] (201 regulatory proteins are currently available from the 314 predicted [22]). Second, a significant number of gene-expression datasets for Escherichia coli is available in the Gene Expression Omnibus database [23].

We downloaded the 10 largest series of gene expression data for E. coli K12 from the Gene Expression Omnibus [24]. Table 2 lists the series identifiers. For data homogeneity, we limited ourselves to Affymetrix microarray platforms only; particularly, the *GeneChip*^Ⓡ^* E. coli Antisense Genome array* and the *GeneChip*^Ⓡ^* E. coli Genome 2.0 array*. Two of the series could possibly confound the experiments because they were used for the development of the RegulonDB; therefore, we excluded them. We verified that the remaining series were not used in the development of RegulonDB.

**Table 2:** List of gene expression series collected from the Gene Expression Omnibus (10 largest series for E. coli K12)

Series id	Platform id	# phenotypes
GSE6836	GPL199	62
GSE33147	GPL199	30
GSE10160-1	GPL199	9
GSE10160-2	GPL3154	4
GSE35371	GPL3154	20
GSE21869	GPL199	5
GSE17505	GPL199	10
GSE34023^a^	GPL3154	7
GSE7398^a^	GPL199	8
GSE4778	GPL199	4

The series marked with ^a^ were omitted due their involvement in the development of the RegulonDB

Each of the series contains samples corresponding to several phenotypes (see Table 2), i.e., the data instances are partitioned into more than two classes. From these series, we first constructed a pool of binary-class datasets that a SVM algorithm can natively process. In particular, for each of the series and each pair of its phenotypes, we combined the samples pertaining to these phenotypes into a new (binary-class) dataset and added it to the pool. Each dataset in the pool contains 6–10 samples.

Next, we extracted a collection of non-overlapping *testing* datasets from this pool as follows. We started by randomly choosing the first dataset, and then repeatedly added a random dataset sharing no phenotype-class (and therefore no sample) with the already included datasets, until such datasets are exhausted. The resulting 71 testing are intended for the final statistical comparisons.

Lastly, we also extracted an auxiliary collection of 100 *selection* datasets which may overlap mutually but are disjoint from the testing datasets. The selection datasets were drawn at random from the pool. They are intended for selecting the best performing of newly proposed gene set types.

### Experimental protocol

The purpose of the experiments is to assess

- (Question 1) whether classifiers learned with the novel gene sets are more accurate than those learned with the state-of-the-art gene sets and those learned with the conventional gene-level approach, and
- (Question 2) whether the novel sets contain genes more correlated in expression than the state-of-the-art gene sets and than random genes.

Our experimental procedure has three steps.

First, we select the best performing of the newly proposed gene set types. We select one type from the three types of operon-based gene sets (OPR, TU, COPR) and one type from the three transcription-factor-based gene sets (TF, REG, SREG). We measure the average classification accuracy estimated by leave-one-out cross-validation on the selection datasets, and the gene set types are ranked using the sum of ranks from the Friedman’s rank-sum test. This prior selection step is employed to reduce the number of statistical tests conducted on the testing datasets.

Second, we address Question 1 above. In particular, we evaluate the performance of the selected gene sets on the testing datasets against the baseline gene sets (see paragraph *Baseline gene sets* above) and against the conventional gene-level approach using leave-one-out cross-validation and the one-sided paired Wilcoxon test. Recall that the testing datasets are independent of the selection data sets. For learning the conventional gene-level classifiers, we use the set of genes corresponding to the union of all the gene sets of the type the conventional classifier is compared to. This is to make sure that both the compared approaches receive the same amount of information on their inputs.

Third, we visit Question 2 in that we evaluate the correlation of expression of genes in the selected novel gene sets in comparison to the state-of-the-art gene sets and to random genes. We employ a sampling based approach for scalability. In particular, we sample 5000 pairs of genes, calculate the expression correlation coefficient for each sampled pair on all the available data sets, and plot the resulting densities of the obtained coefficients. More precisely, for each gene set type the following two steps are iterated 5000 times: (i) randomly select a gene set of the given type, with probability corresponding to the number of 2-combinations of its size, (ii) from the selected gene set, randomly select a pair of two distinct genes and compute their correlation coefficient. We omit single-gene sets. The procedure to calculate the correlation histogram for random gene pairs (not bound to any gene set) is similar, except the gene pairs are sampled from among all genes rather than a (sampled) gene set.

## Results and discussion

### Selection

The results obtained on the 100 selection data sets are shown in Table 3. The best performing types of gene sets in terms of the sum-of-ranks criterion (here co-inciding also with the mean-accuracy criterion) are the Continuous Operon Subsequence (COPR) type out of the operon-based family, and the Regulon (REG) type out of the transcription-factor-based family.

**Table 3:** Results obtained with the newly proposed gene sets on the selection data sets

Gene-set type	Mean accuracy [%]	Sum of ranks
Operon (OPR)	81.50	196.00
Transcriptional unit (TU)	82.17	198.50
Continuous subsequence (COPR)	*83.00*	*205.50*
Transcription factor (TF)	78.69	179.00
Regulon (REG)	*82.67*	*211.50*
Strict regulon (SREG)	82.50	209.50

Columns contain the mean accuracies and sum-of-ranks indicators over the datasets, higher rank indicates better performance. Here, the best ranked gene-set types from the two categories (operon-based, transcription-factor based) are *COPR* and *REG*, respectively

Therefore we select the two mentioned gene set types for the subsequent comparison with state-of-the-art gene sets and conventional (gene-level) classification on the independent testing data sets.

### Classification

Table 4 presents the main statistical findings. The results are based on the counts of wins, ties, and losses on the 71 testing data sets for each pair of tested methods^1^; these counts are detailed in the Additional file 1. The compared variants include the two selected novel gene-set types (COPR, REG), the state-of-the-art gene-set type (GO+KEGG) as well the randomized and gene-level variants of the former three methods. As mentioned already, the gene-level variant of a method works with features corresponding to genes from the union of the gene sets pertaining to that method. In other words, the gene sets are ‘dissolved’ into individual genes. In the special case of COPR, this union in fact contains *all* genes from the original data sets because every gene of Ecoli K12 is associated to an operon.

**Table 4:** Summary of the main experimental findings

Control	Novel sets		Conventional sets
	COPR sets	REG sets	KEGG+GO sets
Randomized	*↑* better	*↑* better	*↑* same (*p*=1)
	(*p*=0.02950)	(*p*=0.0186)	
Gene-level	*↑* better	*↑* better	*↑* worse (*p*=0.00254)
	(*p*=0.01776)	(*p*=0.03809)	

Both the selected types of the newly proposed gene sets (i.e., COPR and REG) perform significantly better than their randomized and gene-level versions. On the contrary, the state-of-the-art gene set type (KEGG+GO) performs indistinguishably from its randomized version and significantly worse than its gene-level version. As detailed in main text, the *p*-values correspond to the one-sided paired Wilcoxon test applied on the win/tie/loss counts determined by leave-one-out cross-validation of predictive accuracies

The observations confirm the main hypothesis of our study, that is, the selected newly proposed gene sets based on regulation-interaction information (COPR, REG) significantly outperform their randomized counterparts as well as their gene-level counterparts. This is however not the case for the conventional gene-set type (KEGG+GO), confirming the observations of the recent line of research [1, 2, 5, 6].

### Correlation

**Fig. 3:**
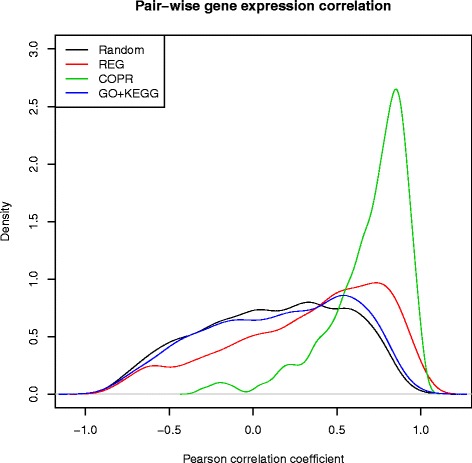
Density plots of pair-wise gene expression correlations. Random: each two genes are randomly sampled from among all genes. Remaining plots: a gene-set is first sampled from a given category (GO+KEGG, REG, COPR), and the two genes are then sampled from that set

Our explanation for the observed favorable results of the new gene-set types in comparison to the state-of-the-art type is that the former collect genes more correlated in terms of expression. Here we validate this hypothesis. Figure 3 shows the density plots induced on histograms of pair-wise gene expression correlation coefficients for random gene pairs, and for pairs sampled jointly from a random gene set of the given type. The types include the state-of-the-art gene-set type (KO+KEGG) and the two selected novel types (REG, COPR).

As could be anticipated, the histogram pertaining to random gene pairs is almost symmetrical around the mean correlation 0. The GO+KEGG histogram is only slightly skewed towards the right, indicating a weak positive correlation trend. The REG histogram is significantly more skewed to the positive correlation, and the COPR histogram even more so. These observations confirm the assumption that the novel gene set types are more correlated than the conventional type.

### Additional comparisons

To gain further insights into the performance of the novel gene sets, we performed several additional experiments.

First, we compared predictive accuracy obtained with the REG and COPR gene sets to predictive accuracy obtained with representation based on transcription factors as single genes. While classifiers based on REG and COPR gene sets outperformed classifiers based on transcription factors, which is a result consistent with our expectations that the novel gene sets based on regulons better capture the activity and concentration of the transcription factors, the difference was not statistically significant (REG *p* = 0.15, COPR *p* = 0.25 using one-sided Wilcoxon signed-rank test).

Second, we tested if more advanced aggregation methods could lead to better predictive performance. We repeated the experiments with aggregation based on principal component analysis (PCAgg) and with SetSig aggregation [1]. We found that both PCAgg and SetSig lead to worse predictive accuracy than aggregation by averaging when using the novel gene sets and to statistically insignificant differences for GO+KEGG gene sets. This may be explained by the relatively small sizes of the prokaryotic datasets used in the experiments presented in this paper causing high variance of the PCAgg/SetSig calculations.

Third, we experimented with a method constructing ad-hoc gene sets on training data by a hierarchical clustering algorithm, cutting the resulting dendrograms at such a depth so that the number of clusters would be equal to the number of the respective gene sets. We compared the predictive performance of this method to performance of the classifiers based on COPR and REG gene sets. The method using REG gene sets was significantly better than CLUST (*p* = 0.04 using one-sided Wilcoxon signed-rank test) whereas the method using COPR gene sets was better only insignificantly (*p* = 0.16 using one-sided Wilcoxon signed-rank test). A disadvantage of CLUST compared to our novel gene sets is also that the created clusters often do not have to be biologically meaningful which negatively affects interpretability.

### Extendibility to eukaryotic organisms

The gene sets introduced in this paper were designed primarily for prokaryotic organisms which have their genes organized in operon structures. Since eukaryotic organisms have different and more complicated structure of genomes, not all of our results are directly extendable to eukaryotic organisms. This is true specifically for the gene sets based on the operon structures as we cannot even define the COPR gene sets for eukaryotes. It might be interesting to replace the operon-based family by analogical eukaryotic concepts such as *posttranscriptional operons* [25]. On the other hand, the ideas of the gene sets based on transcription factor regulation can be extended to eukaryotes directly. However, the main problem is that the current knowledge of transcription factor regulatory networks is rather incomplete and therefore the quality of the gene sets based on transcription factors can be expected to be low.

## Conclusions

We evaluated the performance of a new type of gene sets based on the structure of transcription-regulation networks and on the operon structure of bacterial genomes using attribute-value machine learning and gene-set aggregation. All the proposed gene sets are new in the context of predictive classification and are a salient contribution of this paper.

We hypothesized that these new gene sets would collect genes more correlated in expression than the most usual state-of-the-art gene sets based on the gene ontology and KEGG database information, and that they would also enable to construct more accurate classifiers.

Our experiments on prokaryotic gene expression data series from the Gene Expression Omnibus confirm the hypothesis. In particular, the newly proposed gene sets based on regulation-interaction information significantly outperform their randomized counterparts as well as their gene-level counterparts in terms of classification accuracy. This is however not the case for the state-of-the-art gene-set type (KEGG+GO), confirming the observations of the recent line of research [1, 2, 5, 6]. It also turns out that the new gene sets are contain genes with more correlated expression than the state-of-the-art gene sets.

## Endnote

^1^ We base the comparisons on the win/tie/loss count to adhere to the methodology [26] for comparing classifiers on multiple data sets. Demšar[26] specifically deems it incorrect to rank classifiers by averaging accuracies over multiple data sets.

## Additional file

Additional file 1 **Supplementary material.** (ZIP 90 kb)

## Abbreviations

COPR: Continuous operon subsequence
E. coli: Escherichia coli
GO: The gene ontology
KEGG: The Kyoto Encyclopedia of genes and genomes
OPR: Operon
TU: Transcriptional unit
TF: Transcription factor gene sets
REG: Regulon
SREG: Strict regulon
SVM: Support vector machine
