# Supplementary material for: Novel gene sets improve set-level classification of prokaryotic gene expression data
Source: BMC Bioinformatics. 2015 Oct 28;16:348. doi: 10.1186/s12859-015-0786-7 (PMC4625461; doi:10.1186/s12859-015-0786-7)
Supplement: Supplementary file 1 — Supplementary material. (ZIP 90 kb) [file 12859_2015_786_MOESM1_ESM.zip › table_of_phenotypes.pdf]

1 Table of phenotypes

Dataset	Phenotype I	#	Phenotype II	#	Series
1	fis___U_N0075	3	murI__U_N0075	3	GSE6836
2	cspF__U_N0075	3	sulA___U_N0025	3	GSE6836
3	dinI___U_N0025	3	mazF___U_N0025	3	GSE6836
4	dnaT__U_N0075	3	relE___U_N0025	3	GSE6836
5	cpxR__U_N0075	3	crp___U_N0075	3	GSE6836
6	dnaA__U_N0075	3	gyrI__U_N0075	3	GSE6836
7	hscA__U_N0075	3	rstB__U_N0075	3	GSE6836
8	menC__U_N0075	3	T36_N0025	3	GSE6836
9	galF__U_N0075	3	gyrA__U_N0075	3	GSE6836
10	gcvR__U_N0075	3	hlpA__U_N0075	3	GSE6836
11	dam___U_N0075	3	minD__U_N0075	3	GSE6836
12	fklB__U_N0075	3	holD__U_N0075	3	GSE6836
13	era___U_N0075	3	nupC__U_N0075	3	GSE6836
14	lexA___U_N0025	3	luc___U_N0000	3	GSE6836
15	rimI__U_N0075	3	T24_N0000	3	GSE6836
16	dinP___U_N0025	3	luc___U_N0075	3	GSE6836
17	T24_N0025	3	T60_N0025	3	GSE6836
18	lon___U_N0025	3	mcrC__U_N0075	3	GSE6836
19	ldrA__U_N0075	3	recA___U_N0025	3	GSE6836
20	crcB__U_N0075	3	folA__U_N0075	3	GSE6836
21	uspA__U_N0075	3	yebF__U_N0075	3	GSE6836
22	ruvC__U_N0075	3	uvrA___U_N0025	3	GSE6836
23	bcp___U_N0075	3	umuD___U_N0025	3	GSE6836
24	minE__U_N0075	3	pyrC__U_N0075	3	GSE6836
25	b2618_U_N0075	3	yoeB__U_N0075	3	GSE6836
26	T12_N0025	3	T60_N0000	3	GSE6836
27	mcrB__U_N0075	3	nrdA__U_N0075	3	GSE6836
28	ruvA___U_N0025	3	sbcB__U_N0075	3	GSE6836
29	T48_N0025	3	zipA__U_N0075	3	GSE6836
30	dnaN__U_N0075	3	luc___U_N0025	3	GSE6836
31	menB__U_N0075	3	T0_N0025	3	GSE6836
32	Strain glycerol2 day 44	3	Strain lactateE day 60	3	GSE33147
33	Strain glycerolA day 20	3	Wildtype grown on 2g/L glycerol	5	GSE33147
34	Strain lactateD day 20	3	Strain lactateD day 60	3	GSE33147
35	Strain glycerolE day 20	3	Strain lactate2 day 60	3	GSE33147
36	Strain glycerolA day 44	3	Strain lactate3 day 60	3	GSE33147
37	Strain glycerolE day 44	3	Strain lactate3 day 20	3	GSE33147
38	Strain glycerolC day 20	3	Strain glycerolD day 44	3	GSE33147
39	Strain glycerol1 day 20	3	Strain glycerol2 day 20	3	GSE33147
40	Strain lactateA day 20	3	Strain lactateB day 60	3	GSE33147
41	Strain glycerolB day 20	3	Wildtype grown on 2g/L lactate	6	GSE33147
42	Strain glycerolD day 20	3	Strain lactateA day 60	4	GSE33147
43	Strain glycerolC day 44	3	Strain lactateB day 20	3	GSE33147
44	Strain glycerol1 day 44	3	Strain lactateE day 20	3	GSE33147
45	Strain lactate2 day 20	3	Strain lactateC day 20	3	GSE33147
46	Strain glycerolB day 44	3	Strain lactateC day 60	3	GSE33147
47	Ecoli_treated_cefmec_-5min	3	Ecoli_treated_cefmec_20min	3	GSE10160
48	Ecoli_treated_mecillinam0.03_40min	3	Ecoli_untreated_40min	3	GSE10160
49	Ecoli_treated_cefmec_40min	3	Ecoli_untreated_-5min	3	GSE10160
50	Ecoli_treated_cefmec_5min	3	Ecoli_treated_cefsulodin10_40min	3	GSE10160
51	Ecoli_treated_cefsulodin60_10min	3	Ecoli_untreated_60min	3	GSE10160
52	Ecoli_treated_mecillinam0.3_60min	3	Ecoli_untreated_10min	3	GSE10160
53	E_coli_sham_sinusoidal_cont_2.5h	3	E_coli_treated_sinusoidal_interm_15h	3	GSE35371
54	E_coli_sham_powerline_interm_8min	3	E_coli_sham_sinusoidal_cont_8min	3	GSE35371
55	E_coli_negativ_control_left_chamber	3	E_coli_treated_powerline_interm_8min	3	GSE35371
56	E_coli_sham_powerline_interm_15h	3	E_coli_sham_powerline_interm_2.5h	3	GSE35371
57	E_coli_sham_sinusoidal_interm_15h	3	E_coli_sham_sinusoidal_interm_2.5h	3	GSE35371
58	E_coli_sham_sinusoidal_cont_15h	3	E_coli_treated_powerline_interm_2.5h	3	GSE35371
59	E_coli_response_control_water_added	3	E_coli_treated_sinusoidal_cont_2.5h	3	GSE35371
60	E_coli_treated_sinusoidal_cont_15h	3	E_coli_treated_sinusoidal_cont_8min	3	GSE35371
61	E_coli_negativ_control_right_chamber	3	E_coli_treated_sinusoidal_interm_2.5h	3	GSE35371
62	E_coli_response_control_1mM_H2O2_added	3	E_coli_treated_powerline_interm_15h	3	GSE35371
63	YA027	3	YA074	3	GSE21869
64	3-84-4	3	YA105	3	GSE21869
65	Recombinant, high cell density, Time S4, IPTG	3	Wild-type, high cell density, Time S1, no IPTG	3	GSE17505
66	Recombinant, high cell density, Time S1, IPTG	3	Wild-type, high cell density, Time S1, IPTG	3	GSE17505
67	Recombinant, high cell density, Time S0, no IPTG	3	Recombinant, high cell density, Time S4, no IPTG	3	GSE17505
68	Recombinant, high cell density, Time S1, no IPTG	3	Wild-type, high cell density, Time S4, IPTG	3	GSE17505
69	Wild-type, high cell density, Time S0, no IPTG	3	Wild-type, high cell density, Time S4, no IPTG	3	GSE17505
70	Acid shift 1 min	5	Acid shift time 0	5	GSE4778
71	Acid shift 10 min	5	Acid shift 5 min	5	GSE4778

Table 1: List of datasets with phenotypes including number of samples and NCBI series identifier.
