# Supplementary material for: Novel gene sets improve set-level classification of prokaryotic gene expression data
Source: BMC Bioinformatics. 2015 Oct 28;16:348. doi: 10.1186/s12859-015-0786-7 (PMC4625461; doi:10.1186/s12859-015-0786-7)
Supplement: Supplementary file 1 — Supplementary material. (ZIP 90 kb) [file 12859_2015_786_MOESM1_ESM.zip › wins_ties_losses.pdf]

1 Counts of Wins/Ties/Losses

Table 1: Each cell shows the numbers of wins, ties and losses of the method indicated in the row header against the type shown in the column header. The wins, ties and losses are in terms of predictive accuracy estimated by leave-one-out cross-validation on individual data sets from the testing collection. COPR and REG pertain to set-level based classification using the two selected novel gene set types, GO+KEGG represents the state of the art gene sets. The prefix 'R-' indicates the randomized version of the method next to the dash. The prefix 'GL-' indicates the conventional gene-level version of the method.

wins-ties-losses	COPR	R-COPR	GL-COPR	GO+KEGG	R-GO+KEGG
COPR	0-71-0	5-66-0	6-65-0	13-57-1	13-55-3
R-COPR	0-66-5	0-71-0	2-68-1	12-56-3	12-54-5
GL-COPR	0-65-6	1-68-2	0-71-0	12-56-3	12-54-5
GO+KEGG	1-57-13	3-56-12	3-56-12	0-71-0	7-58-6
R-GO+KEGG	3-55-13	5-54-12	5-54-12	6-58-7	0-71-0
GL-GO+KEGG	3-62-6	4-66-1	4-66-1	14-53-4	13-53-5
REG	10-53-8	12-53-6	13-52-6	15-54-2	17-50-4
R-REG	4-58-9	5-61-5	6-59-6	13-53-5	13-51-7
GL-REG	3-60-8	4-62-5	4-62-5	12-55-4	12-53-6

wins-ties-losses (cont'd)	GL-GO+KEGG	REG	R-REG	GL-REG
COPR	6-62-3	8-53-10	9-58-4	8-60-3
R-COPR	1-66-4	6-53-12	5-61-5	5-62-4
GL-COPR	1-66-4	6-52-13	6-59-6	5-62-4
GO+KEGG	4-53-14	2-54-15	5-53-13	4-55-12
R-GO+KEGG	5-53-13	4-50-17	7-51-13	6-53-12
GL-GO+KEGG	0-71-0	6-55-10	7-60-4	5-64-2
REG	10-55-6	0-71-0	11-56-4	9-59-3
R-REG	4-60-7	4-56-11	0-71-0	5-62-4
GL-REG	2-64-5	3-59-9	4-62-5	0-71-0
